# Supplementary material for: OpgH is an essential regulator of Caulobacter morphology
Source: bioRxiv. 2023 Aug 28:2023.08.28.555136. Preprint. [Version 1] doi: 10.1101/2023.08.28.555136 (PMC10491104; doi:10.1101/2023.08.28.555136)
Supplement: Supplement 3 [file NIHPP2023.08.28.555136v1-supplement-3.pdf]

bioRxiv preprint doi: https://doi.org/10.1101/2023.08.28.555136; this version posted August 28, 2023. The copyright holder for this preprint (which was not certified by peer review) is the author/funder, who has granted bioRxiv a license to display the preprint in perpetuity. It is made available under aCC-BY 4.0 International license.

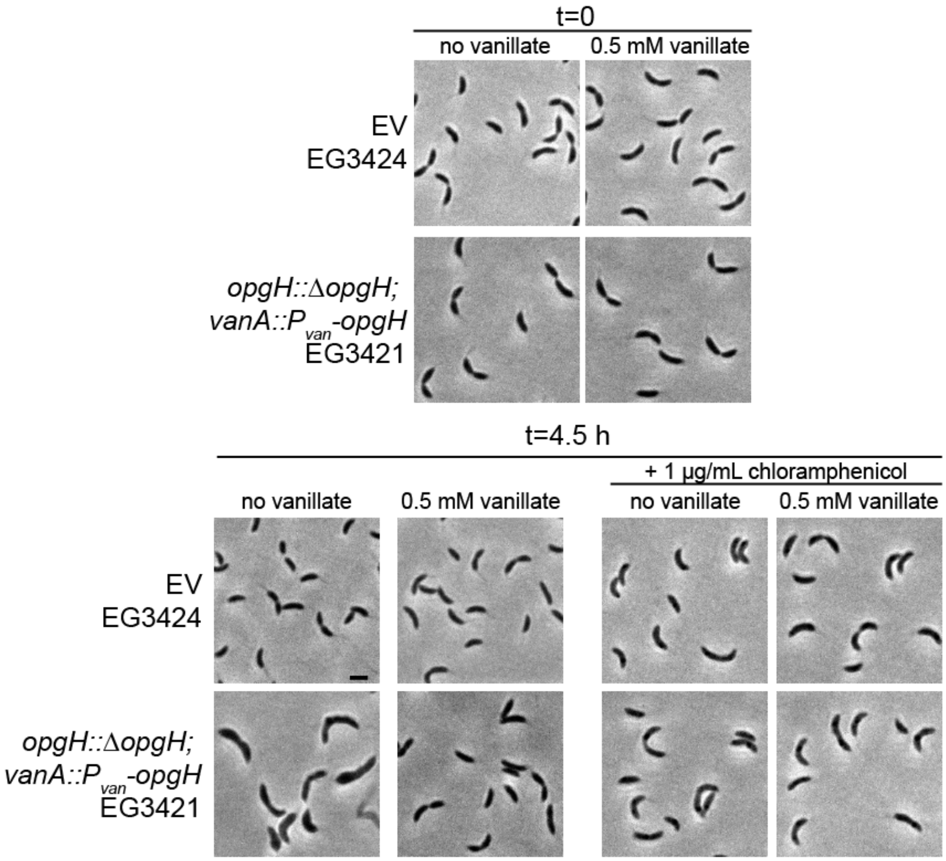

Supplemental Figure 1.

The OpgH depletion phenotype is reliant on cell growth.

Phase contrast images of and empty vector (EV, EG3424) and OpgH depletion (EG3421) strains at the start (t=0) and end (t=4.5 hours) of treatment with sub-lethal chloramphenicol (1 µg/mL) in the presence and absence of vanillate. Scale bar, 2 µm.

bioRxiv preprint doi: https://doi.org/10.1101/2023.08.28.555136; this version posted August 28, 2023. The copyright holder for this preprint (which was not certified by peer review) is the author/funder, who has granted bioRxiv a license to display the preprint in perpetuity. It is made available under aCC-BY 4.0 International license.

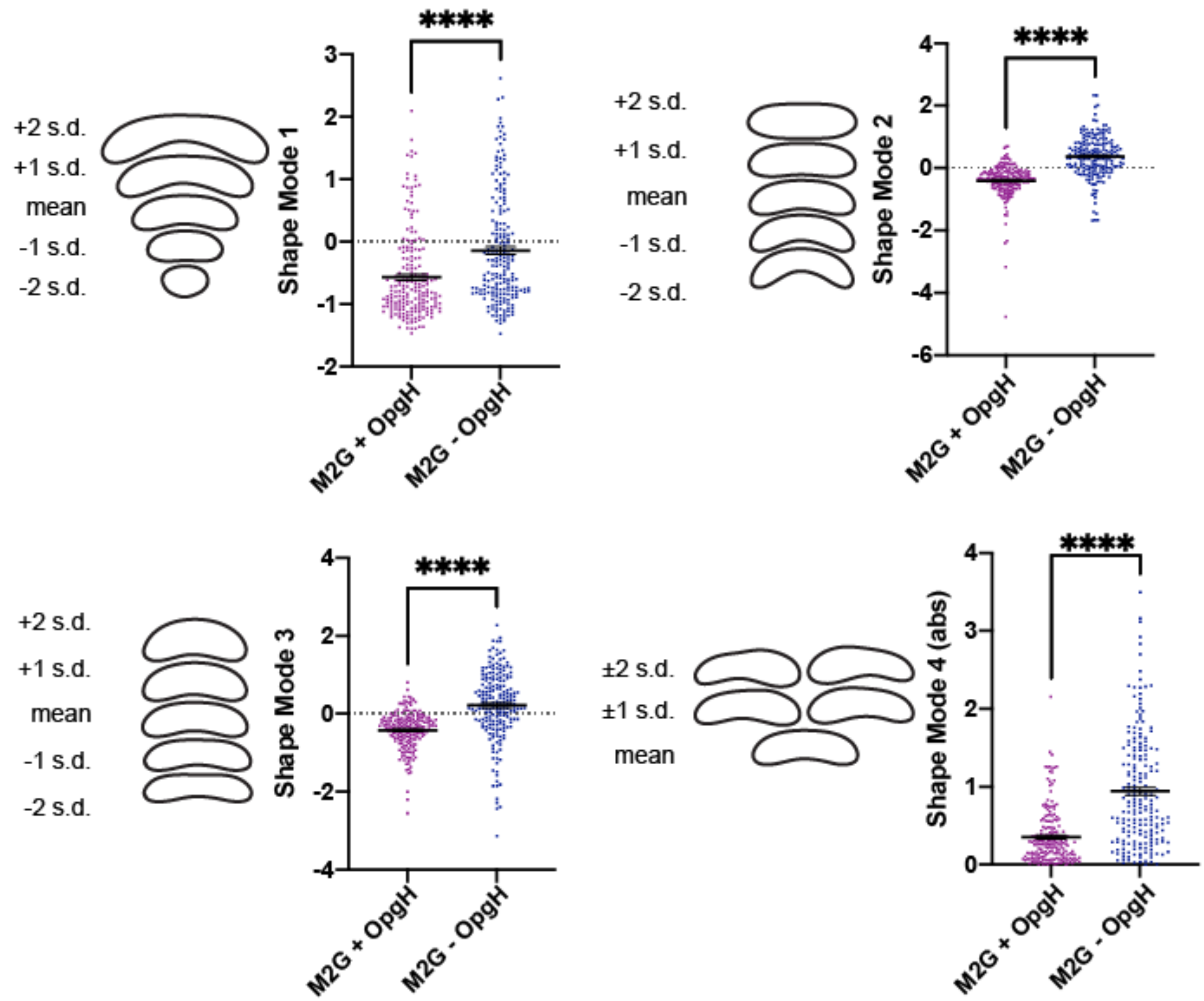

Supplemental Figure 2.

OpgH depletion causes morphological defects in minimal media

Principal component analysis (PCA) of the OpgH depletion strain (EG3421) after 5 hours grown in M2G with 0.5 mM vanillate (purple, +OpgH) or without vanillate (blue, -OpgH). Scatter plots of 200 cells are presented. Shape modes 1, 2, 3, and 4 correspond to length, curvature, width, and asymmetric bulging. Contours indicate the mean shape and 1 or 2 standard deviations from the mean. Shape mode 4 shows the absolute value. Statistical analysis uses a Mann-Whitney unpaired t-test. **** = P < 0.0001.

bioRxiv preprint doi: https://doi.org/10.1101/2023.08.28.555136; this version posted August 28, 2023. The copyright holder for this preprint (which was not certified by peer review) is the author/funder, who has granted bioRxiv a license to display the preprint in perpetuity. It is made available under aCC-BY 4.0 International license.

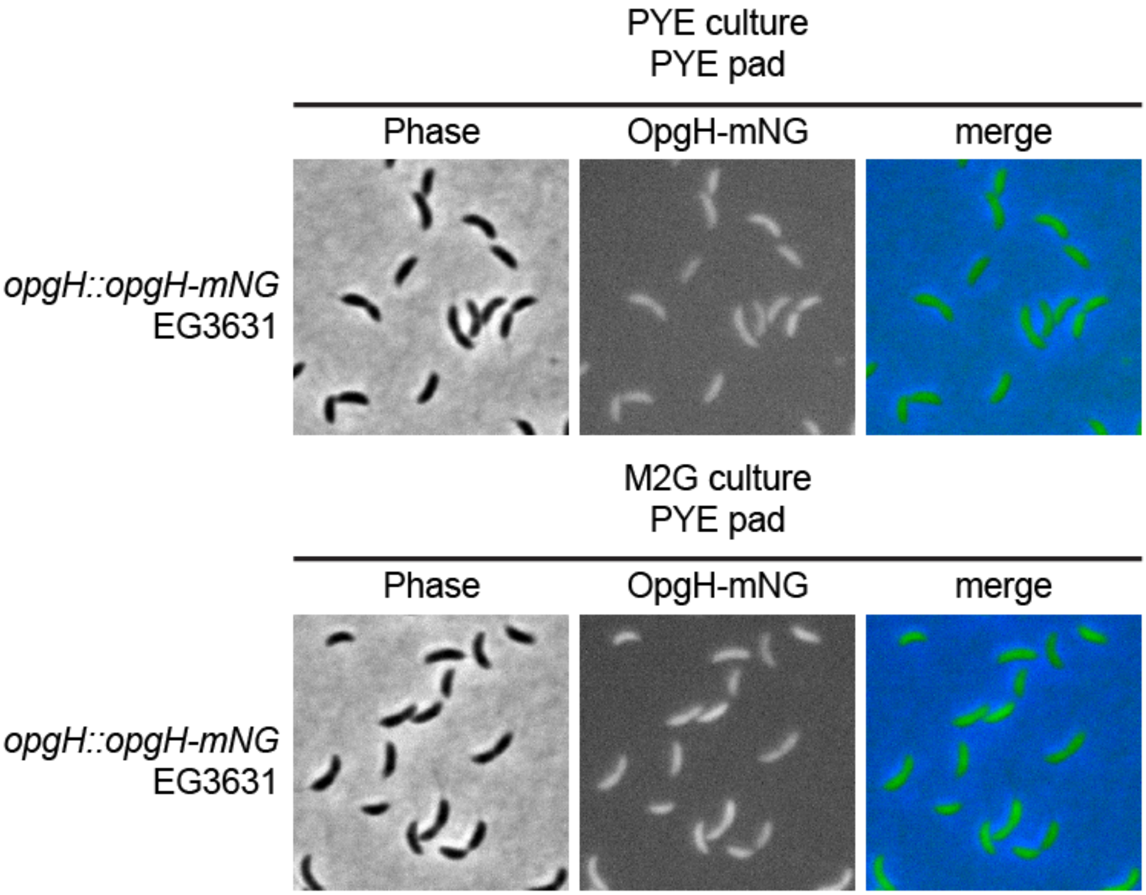

Supplemental Figure 3.

OpgH is diffuse within the cell.

Phase contrast, epifluorescence, and merged images showing localization of OpgH with a C-terminal mNeonGreen (mNG) tag (EG3631) grown in PYE or M2G.

bioRxiv preprint doi: https://doi.org/10.1101/2023.08.28.555136; this version posted August 28, 2023. The copyright holder for this preprint (which was not certified by peer review) is the author/funder, who has granted bioRxiv a license to display the preprint in perpetuity. It is made available under aCC-BY 4.0 International license.

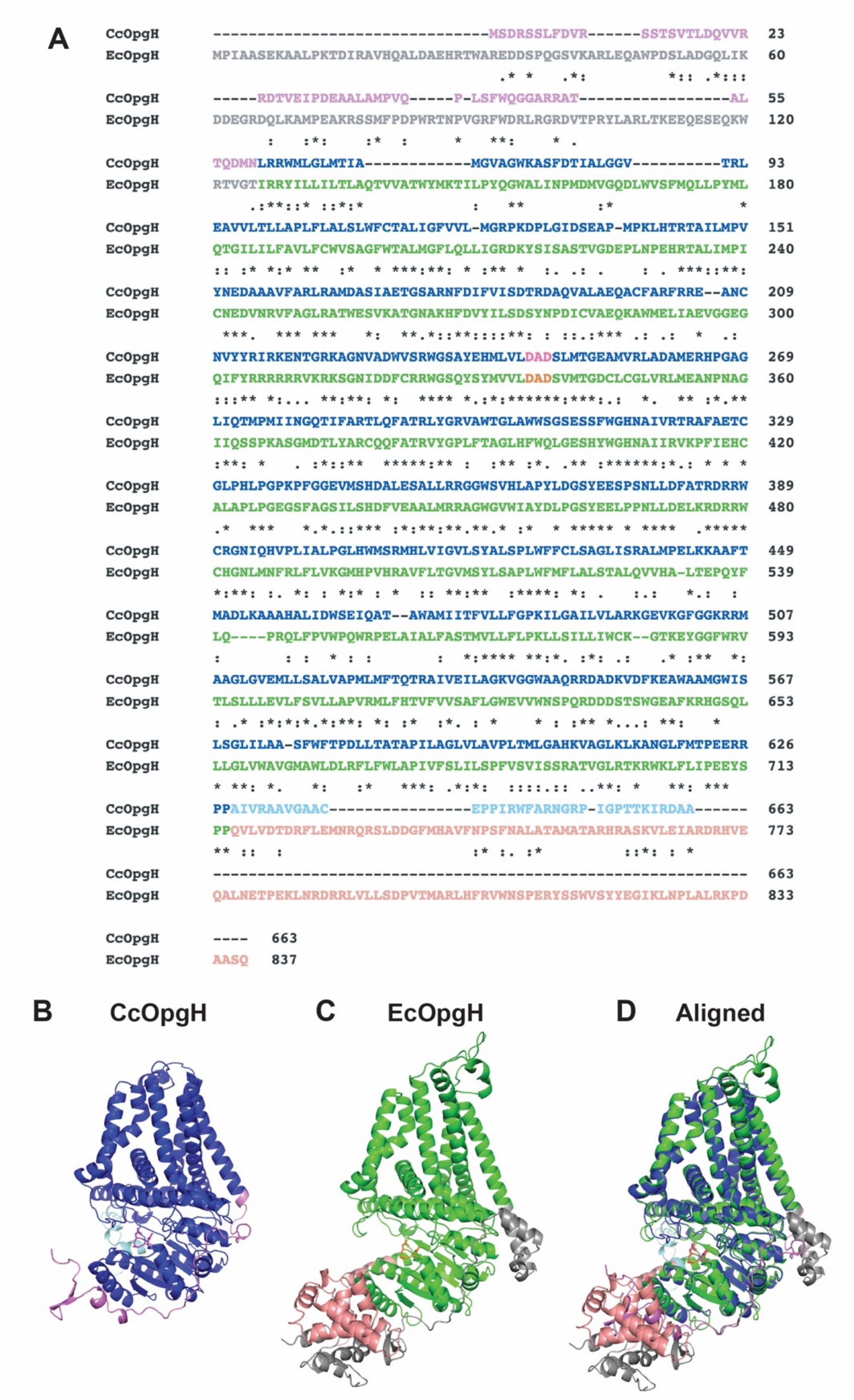

Supplemental Figure 4.

Sequence and structure alignments of *E. coli* and *Caulobacter* OpgH reveal similarities and differences.

bioRxiv preprint doi: https://doi.org/10.1101/2023.08.28.555136; this version posted August 28, 2023. The copyright holder for this preprint (which was not certified by peer review) is the author/funder, who has granted bioRxiv a license to display the preprint in perpetuity. It is made available under aCC-BY 4.0 International license.

A. The primary sequences of *Caulobacter* (CcOpgH) and *E. coli* (EcOpgH) were aligned Clustal Omega. The poorly conserved N- and C-terminal regions of each are colored independently from the well-conserved central region. The predicted catalytic D-A-D residues of each are highlighted in magenta (CcOpgH) and orange (EcOpgH). **B** and **C**. Alphafold predicted structures of CcOpgH (B) and EcOpgH (C) with residues colored as in (A) and oriented with the predicted periplasmic face at the top and cytoplasmic regions at the bottom. **D.** The structures in B and C were aligned in Pymol yielding an rmsd of 0.678. Major differences observed are in the N- and C-termini, as well as in predicted periplasmic loops of EcOpgH.

bioRxiv preprint doi: https://doi.org/10.1101/2023.08.28.555136; this version posted August 28, 2023. The copyright holder for this preprint (which was not certified by peer review) is the author/funder, who has granted bioRxiv a license to display the preprint in perpetuity. It is made available under aCC-BY 4.0 International license.

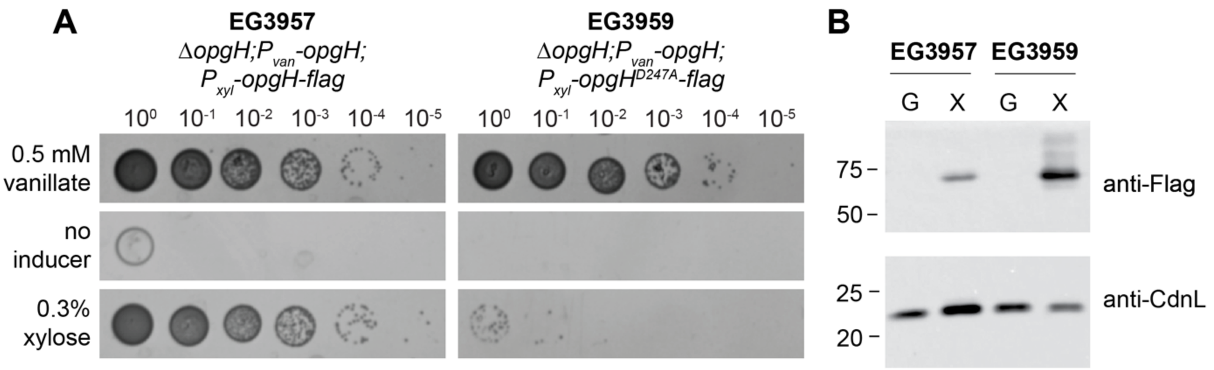

Supplemental Figure 5.

3X-Flag-tagged $OpgH_{D247A}$ cannot complement loss of OpgH but is stably produced.

A. Spot dilutions of the OpgH depletion strain (Δ*opgH* + P_{van}-*opgH*) with xylose inducible WT *opgH-3X-Flag* (EG3957) or *opgH*$_{D247A}$*-3X-Flag* (EG3959) grown on M2G with indicated inducer for two days. **B.** Immunoblot of lysates from the indicated strains grown in PYE with glucose (G) or xylose (X) for 6.5 hours. CdnL was used as a loading control.

bioRxiv preprint doi: https://doi.org/10.1101/2023.08.28.555136; this version posted August 28, 2023. The copyright holder for this preprint (which was not certified by peer review) is the author/funder, who has granted bioRxiv a license to display the preprint in perpetuity. It is made available under aCC-BY 4.0 International license.

Supplemental Table 1.

Polar metabolites in extracts from cells producing OpgH or depleted of OpgH for 5 h in M2G or PYE. Metabolites reduced at least two-fold during OpgH depletion in both media conditions are highlighted in red, those increased at least two-fold during OpgH depletion in both media conditions are highlighted in green.

Supplemental Table 2.

Strains and plasmids used in this study.
